# Histopathological Patterns in Mycosis Fungoides: A Cross-Sectional Study

**DOI:** 10.1155/jskc/9995443

**Published:** 2025-10-14

**Authors:** Fatemeh Mohaghegh, Mina Shayan, Parvin Rajabi, Farahnaz Fatemi Naeini, Zahra Nikyar

**Affiliations:** ^1^Department of Dermatology, Skin Diseases and Leishmaniasis Research Center, School of Medicine, Isfahan University of Medical Sciences, Isfahan, Iran; ^2^Department of Pathology, School of Medicine, Isfahan University of Medical Sciences, Isfahan, Iran; ^3^Department of Dermatology, Razi Hospital, Tehran University of Medical Sciences (TUMS), Tehran, Iran

**Keywords:** cytological atypia, eosinophils, epidermotropism, histopathology, Mycosis fungoides

## Abstract

**Introduction:**

Mycosis fungoides (MF) represents the most prevalent form of cutaneous lymphoma, characterized by diverse histopathological patterns. However, recent comprehensive studies systematically evaluating the spectrum of histopathological patterns and changes associated with MF in patients within Iran are notably lacking.

**Method:**

This cross-sectional descriptive study analyzed 64 skin biopsy slides diagnosed with MF, archived from 2013 to 2023 at Al-Zahra Hospital and a private dermatopathology laboratory in Isfahan, Iran. Inclusion criteria included all slides with complete demographic data and a confirmed MF diagnosis. Patterns and finding of these slides were analyzed according to age, gender, and biopsy site. Data analysis was performed using SPSS Version 26.0, with significance set at *p* < 0.05.

**Results:**

The study included 31 females and 33 males, with a mean age of 44.64 ± 14.91 years. The most common biopsy site was the trunk (45.31%). The predominant histopathologic patterns observed were psoriasiform (33 cases), lichenoid (14 cases), and spongiotic (10 cases), with universal epidermotropism across all slides. Parakeratosis and cytological atypia were found in 37 and 31 cases, respectively. Notably, eosinophils were more frequent in males (*p*=0.016), and cytological atypia were significantly more common in males than females (*p*=0.044). Mild dermal infiltrate was the most prevalent, particularly in the 35–42 and 43–56 age groups, with a significant age-related variation (*p*=0.041).

**Conclusion:**

This study reveals that psoriasiform, lichenoid, and spongiotic patterns are the most common in MF, with epidermotropism present in all cases. Age and gender significantly influence certain features, but overall, histopathological patterns showed no significant variation by biopsy location or age group.

## 1. Introduction

Mycosis fungoides (MF) represents the most frequently occurring subtype of cutaneous T-cell lymphomas (CTCLs), which constitute a varied group of non-Hodgkin lymphomas derived from T-cells, predominantly presenting in the skin and accounting for approximately half of all CTCL cases [1, 2]. The disease is distinguished by the malignant expansion of CD4+ T cells with a tendency to infiltrate the epidermis, generally exhibiting a slow and prolonged clinical progression. In the early phases, MF commonly appears as red patches or plaques, which can remain for extended periods, often years, without significant progression or affecting the patient's lifespan [3, 4]. In some cases, patients go on to develop skin tumors, which can be followed by the spread to lymph nodes and, in rare instances, to internal organs. This progression is a sign of advanced-stage disease [5]. Diagnostic guidelines for MF have been established by various professional societies, and the methods are generally showing high consistency across these guidelines [6–8]. Diagnosis usually involves a comprehensive evaluation of pathological and immunohistochemical examination, clinical manifestation, and clinical progression, and sometimes molecular biological analysis. Despite this thorough approach, diagnosing MF, particularly in its early stages, remains challenging [9, 10]. Differentiating early MF from benign inflammatory disorders (BIDs) like atopic dermatitis (AD), chronic eczema, and psoriasis is difficult because several clinical and pathological characteristics in MF closely resemble those of BIDs [9, 11]. The absence of cancer cell–specific markers and the limited sensitivity and specificity of genetic assessments for recognizing tumor cell clonality add further complexity to the differential diagnosis. Accurate diagnosis of early MF is crucial for selecting an appropriate therapeutic strategy [5, 12]. Misdiagnosis as BIDs has led to numerous MF cases with unfavorable or even fatal outcomes due to inappropriate treatments, such as the use of immunosuppressants [13, 14].

Histopathological examination of MF patient biopsies can reveal various features, including Pautrier's microabscesses, epidermotropism, larger epidermal lymphocytes compared with dermal lymphocytes, papillary dermal fibrosis, lichenoid reaction, granulomas, infiltration of cytological atypia (hyperchromasia and irregular nuclear membranes) in eccrine glands, hair follicles by cytological atypia, and follicular mucinosis. These characteristics can appear with varying prevalence in different slides [15, 16].

In recent years, few studies have examined the histopathological patterns and changes in MF patients in Iran. Such research is vital for increasing the knowledge and awareness of doctors and specialists about MF, its various patterns, and findings, thereby paving the way for more effective diagnostic and therapeutic measures.

## 2. Method

### 2.1. Study Design

This cross-sectional descriptive study examined skin biopsy slides from 2013 to 2023, archived at two pathology centers: Al-Zahra Hospital (a major tertiary care and educational hospital affiliated with Isfahan University of Medical Sciences) and a Dr. Rajabi's Private Dermatopathology Laboratory, an independent diagnostic center directed by Dr. Parvin Rajabi, a faculty member in the Department of Pathology, School of Medicine, Isfahan University of Medical Sciences. Inclusion criteria encompassed pathologic slides from skin biopsies diagnosed with MF. Exclusion criteria included slides that were too distorted to review or interpret and cases with incomplete demographic information.

### 2.2. Sample Size

Based on the population of MF patients with archived skin biopsy samples at the Pathology Laboratory Center of Al-Zahra Hospital and the Referral Dermatopathology Laboratory in Isfahan, and applying a 95% confidence level with a 5% margin of error, the required sample size for this study is estimated to be 64.

### 2.3. Study Protocol

Following the approval of the Ethics Committee of Isfahan University of Medical Science (code: IR.MUI.MED.REC.1401.129) and obtaining informed consent, 64 pathologic slides were reexamined by a dermatopathologist. Both Al-Zahra Hospital and Dr. Rajabi's Private Dermatopathology Laboratory operate under academic and ethical oversight of the university. Therefore, no additional ethical approval was required from the private laboratory. Prior to inclusion in the research, all patient samples received thorough immunohistochemistry (IHC) assessments, encompassing T-cell receptor (TCR) analysis and CD4/CD8 ratio assessments, to validate the diagnosis of MF. Only patients with definite confirmation of MF based on these IHC data were incorporated into the research.

### 2.4. Histopathological Examination

The main histopathological patterns assessed included the following:

• Spongiotic pattern
• Lichenoid pattern
• Granulomatous pattern
• Psoriasiform pattern
• Vesiculobullous pattern
• Vasculopathic pattern

The histopathological changes examined were as follows:

• Epidermotropism
• Spongiosis
• Parakeratosis
• Intensity of papillary dermal infiltration
• Basal vacuolar damage
• Folliculotropic pattern
• Syringotropic pattern
• Pautrier's microabscess
• Mucinous change in hair follicle
• Granuloma formation
• Perivascular superficial infiltrate
• Pigment incontinence
• Cytological atypia
• Large Cell Transformation.

These items were recorded as present or absent in each sample. These findings were then analyzed in relation to the patient's age at the time of diagnosis, sex, and the location of the biopsy.

The data were analyzed using the Statistical Package for the Social Sciences (SPSS), Version 26.0 (SPSS Inc., Chicago, IL, USA). Descriptive and frequency statistics were computed for the variables under investigation, with statistical significance determined at a *p* value > 0.05. The study protocols adhered to the ethical standards set forth by the Isfahan University of Medical Science Ethics Committee (IR.MUI.MED.REC.1401.129).

## 3. Results

In total, we examined 64 (out of 129 slides which some were redacted due to the exclusion criteria) slides including 31 females and 33 males. A total of 29 slides were from trunk area, 21 from lower limbs, 12 from upper limbs, 1 from facial area, and 1 from scalp. The mean age of the patients was 44.64 ± 14.91 years. The 43–56 age range had the highest number of patients, with 9 males (14.06%) and 8 females (12.5%), totaling 17 patients (26.56%) (*p*=0.446). Most biopsy locations were seen in the trunk, with 14 men (21.88%) and 15 females (23.44%) for 29 patients (45.31%) (*p*=0.5) (Table 1).

The predominant histological pattern was psoriasiform (33 cases, proportion 51.60%) followed by lichenoid and spongiotic (as the latter two patterns were only 21.87% and 15.62% respectively).

All patients in this study were in the early stages of MF (patch and plaque phases), with 100% exhibiting epidermotropism. No differentiation between patch and plaque stages was made. Importantly, no tumoral stage cases were included in this study, as the focus was exclusively on early-stage disease. Also, parakeratosis was found in 37 cases and cytological atypia in 31 cases. Rare patterns included syringotropic (2 cases) and mucinous hair follicle alteration (1 case). Notably, no slides exhibited granulomatous, vasculopathic, or vesiculobullous patterns, and granulomas were absent in all samples. The histopathological examination revealed seven cases displaying two distinct histopathological patterns. Of these, three cases exhibited both spongiotic and psoriasiform patterns, two cases demonstrated a combination of spongiotic and lichenoid patterns, and two cases showed the coexistence of lichenoid and psoriasiform patterns. Notably, two slides exhibited all three histopathological patterns simultaneously.

Epidermotropism was highest in 33 men (51.56%) and 31 females (48.44%). Parakeratosis was common in 20 men (31.25%) and 17 females (26.56%) (*p*=0.641). Syringotropic pattern and mucinous hair follicle alteration were rare, detected in just 1 female (1.56%) and missing in males (*p*=0.964 and *p*=0.298, respectively). Significant differences were reported in eosinophils (8 men (12.5%) compared to 1 female (1.56%) (*p*=0.016), and cytological atypia (20 males (31.25%) compared with 11 females (17.19%)) (*p*=0.044) (Table 2).

Psoriasiform patterns were most frequently identified on the trunk (23.44%), while spongiosis was similarly distributed between the trunk (15.63%) and lower limbs (12.5%). Parakeratosis was prevalent on the trunk (21.88%) and the lower limbs (20.31%). Rare patterns such as the syringotropic pattern were observed equally on the trunk and hand (1.56% each). Notably, certain patterns, such as mucinous change in hair follicles, were only identified in a single biopsy from the trunk (1.56%). Overall, the distribution of histopathological patterns showed no significant differences based on biopsy location.

Psoriasiform patterns were most common in the 43–56 age group (18.75%), while spongiosis was fairly evenly distributed across age ranges, showing no significant differences (*p*=0.763). Parakeratosis was also consistent across age groups, with the highest occurrence in the 43–56 and 57–75 age ranges (17.19% each), although this did not reach statistical significance (*p*=0.209). The syringotropic pattern was rare, appearing in only 1.56% of cases in the 43–56 and 57–75 age groups. Notably, the presence of eosinophils was distributed across all age groups, with no significant age-related variation (*p*=0.636).

Mild dermal infiltrate was the most common, seen in 31.25% of males and 32.81% of females (*p*=0.056). It was most frequent on the trunk (29.69%), followed by the lower limbs (15.63%) (*p*=0.096). Age wise, mild intensity was predominant, especially in the 35–42 and 43–56 age groups (20.31% each), showing a significant difference by age (*p*=0.041). Also, the most prevalent intensity of upper dermal infiltrate was mild, observed across various biopsy sites.

## 4. Discussion

In this study, 64 biopsy slides from patients with a mean age of 44.64 years were analyzed, with the highest number of cases (26.56%) in the 43–56 age range. The most common biopsy location was the trunk (45.31%). Psoriasiform patterns were the most frequently observed histopathologic feature (33 cases), followed by lichenoid (14 cases) and spongiotic patterns (10 cases). Epidermotropism was universally present across all samples, while parakeratosis and cytological atypia were found in 37 and 31 cases, respectively. Rare patterns like syringotropic and mucinous hair follicle alterations were identified in a small number of cases. No granulomatous, vasculopathic, or vesiculobullous patterns were observed. In our study, seven cases exhibited two distinct histopathological patterns, with the most common combination being spongiotic and psoriasiform patterns. Additionally, some cases demonstrated spongiotic and lichenoid patterns, as well as lichenoid and psoriasiform patterns. Notably, two cases displayed all three patterns concurrently. These findings highlight the complexity and overlap of histopathological features in certain dermatological conditions, emphasizing the importance of comprehensive microscopic evaluation for accurate diagnosis. Significant gender differences were noted in the presence of eosinophils and cytological atypia, with higher frequencies in males. The distribution of histopathological patterns showed no significant variation based on biopsy location. Age wise, mild dermal infiltrate was most prevalent in the 35–42 and 43–56 age groups, with a significant age-related difference (*p*=0.041).

The epidermotropism is believed to result from the local intraepidermal environmental conditions, which act as an attractant for neoplastic immune cells [17]. This indicates that epidermotropism is a sensitive, but not specific, measure. The findings of our study align with those of Smoller et al. [18] and Safee Naraghi et al. [19], suggesting that epidermotropism is a critical characteristic for the early identification of MF. It is important to recognize that differentiating early-stage MF from other inflammatory dermatoses presents challenges because of the overlapping histopathological characteristics. In these instances, it may be essential to conduct repeat biopsies and perform meticulous clinicopathological correlation to achieve a precise diagnosis [20]. Epidermotropism, which refers to the infiltration of neoplastic T cells in the epidermis, out of proportion with spongiosis, is a distinctive trait of MF. This feature is commonly observed in both the patch and plaque phases of MF. Accurate identification and comprehension of epidermotropism are essential for diagnosing and managing MF. This requires employing modern histopathologic, immunophenotypic, and genetic approaches to distinguish MF from other epidermotropic illnesses [21, 22].

The presence of spongiosis in MF has been a topic of debate. Previous studies have reported slight spongiosis in 38% of patch/plaque stage lesions and moderate spongiosis in 17% of the cases, without microvesiculation.

A spongiotic histopathological pattern was detected in 16% of our MF biopsies. This prevalence is consistent with earlier descriptions of the spongiotic variant of MF [23] and underscores that, while spongiosis is classically associated with benign eczematous dermatoses, it can also be encountered in MF. Differentiating MF from other spongiotic disorders needs more consideration. A band-like lymphocytic infiltrate in the upper dermis serves as a vital clue. This histological pattern supports an accurate diagnosis and guides the choice of treatment [24]. Cytological atypia (20 males (31.25%) compared with 11 females (17.19%) and eosinophils (8 men (12.5%) compared with 1 female (1.56%) were significantly different (p = 0.044 and *p*=0.016, respectively). The degree of cytological atypia in MF differs based on the disease stage. In the early (patch) stage, atypia can be minimal or even not visible, which makes diagnosis more difficult. As the disease progresses, atypical cells become more noticeable. These cells often show epidermotropism, meaning they migrate into the epidermis and may form groups known as Pautrier's microabscesses [25, 26]. Also, other studies presented that approximately 9%–10% of patients exhibited cytological atypia, making it an unreliable characteristic for diagnosing early lesions. Moreover, the diagnostic value of atypical lymphocytes in initial lesions remains controversial, with varying results reported across different studies (for over three decades) [27–31]. Eosinophils, although not commonly linked with MF, might become conspicuous in specific instances, frequently adding complexity to the clinical presentation. In a unique instance of folliculotropic MF with eosinophilic pneumonia, the levels of eosinophils were notably increased, suggesting a potential interplay between the cancerous cells and the immune system [32]. A further investigation discovered that individuals with MF may experience eosinophilic infiltrates that resemble eosinophilic cellulitis, known as Wells syndrome [33]. This suggests that activated T cells originating from MF might potentially initiate eosinophilic responses.

Regarding age, the majority of the cases showed mild dermal infiltrate, particularly in the 35–42 and 43–56 age groups (20.31% each). There was a noticeable variation in intensity based on age, with a statistically significant difference (*p*=0.041). However, given the limited number of cases, these results should be interpreted with caution. The observed differences may be influenced by other factors or the small sample size, and further research with a larger sample size is needed to confirm these findings.

## 5. Conclusion

This cross-sectional study aimed to investigate the histopathological patterns in MF, providing valuable insights into the disease's presentation across different demographics. The analysis of 64 biopsy slides revealed that the most prevalent histopathologic patterns were psoriasiform, lichenoid, and spongiotic, with universal epidermotropism. The trunk emerged as the most frequent biopsy site, though no significant differences in pattern distribution were observed based on location. Age and gender were found to influence specific histopathological features, with eosinophils and cytological atypia more commonly seen in males. Mild dermal infiltrate was predominant, especially in the 35–56 age range, suggesting potential age-related variations in disease expression. The study highlights the heterogeneity of MF and underscores the importance of considering patient demographics in histopathological evaluation. These findings contribute to a deeper understanding of the disease and support the need for further research with larger sample sizes to validate the observed patterns and their clinical relevance.

## Figures and Tables

**Table 1:** Demographic and clinical characteristics of patients.

		**Male**	**Female**	**Total**	**p** **value**

Age		47.9394 ± 15.58232	41.1290 ± 13.54435	44.6406 ± 14.91496	0.408

Age range	15–34	6 (9.375)	10 (15.625)	16 (25)	0.446
	35–42	8 (12.5)	8 (12.5)	16 (25)	
	43–56	9 (14.0625)	8 (12.5)	17 (26.5625)	
	57–75	10 (15.625)	5 (7.8125)	15 (23.4375)	

Biopsy location	Trunk	14 (21.875)	15 (23.4375)	29 (45.3125)	0.5
	Hand	8 (12.5)	4 (6.25)	12 (18.75)	
	Leg	10 (15.625)	11 (17.1875)	21 (32.8125)	
	Scalp	1 (1.5625)	0 (0)	1 (1.5625)	
	Face	0 (0)	1 (1.5625)	1 (1.5625)	

Dermal infiltrate	Mild	20 (31.25)	21 (32.8125)	41 (64.0625)	0.056
	Moderate	4 (6.25)	8 (12.5)	12 (18.75)	
	Severe	9 (14.0625)	2 (3.125)	11 (17.1875)	

*Note:* The data are presented as the number of cases (%). Chi-square tests were used to calculate the *p* values, which indicate the statistical significance of differences observed between groups for each pattern.

**Table 2:** Distribution of histopathological patterns in MF by sex, biopsy location, and age range.

Histopathological patterns		Sex		*p* value	Biopsy location					*p* value	Age range				*p* value
		Male	Female		Trunk	Hand	Leg	Scalp	Face		15–34	35–42	43–56	57–75	
Dermal infiltrate	Mild	20 (31.25)	21 (32.8125)	0.056	19 (29.6875)	7 (10.9375)	10 (15.625)	0 (0)	0 (0)	0.096	10 (15.625)	13 (20.3125)	13 (20.3125)	5 (7.8125)	**0.041**
	Moderate	4 (6.25)	8 (12.5)		7 (10.9375)	1 (1.5625)	3 (4.6875)	0 (0)	1 (1.5625)		5 (7.8125)	1 (1.5625)	2 (3.125)	4 (6.25)	
	Severe	9 (14.0625)	2 (3.125)		3 (4.6875)	4 (6.25)	3 (4.6875)	1 (1.5625)	0 (0)		1 (1.5625)	2 (3.125)	2 (3.125)	6 (9.375)	

Spongiosis		14 (21.875)	9 (14.0625)	0.264	10 (15.625)	3 (4.6875)	8 (12.5)	1 (1.5625)	1 (1.5625)	0.372	4 (6.25)	6 (9.375)	7 (10.9375)	6 (9.375)	0.763

Parakeratosis		20 (31.25)	17 (26.562)	0.641	14 (21.875)	8 (12.5)	13 (20.3125)	1 (1.5625)	1 (1.5625)	0.546	6 (9.375)	9 (14.0625)	11 (17.1875)	11 (17.1875)	0.209

Basal vacuolar damage		4 (6.25)	6 (9.375)	0.426	6 (9.375)	1 (1.562)	3 (4.6875)	0 (0)	0 (0)	0.836	3 (4.6875)	1 (1.5625)	2 (3.125)	4 (6.25)	0.429

Folliculotropic pattern		8 (12.5)	5 (7.8125)	0.42	4 (6.25)	2 (3.125)	6 (9.375)	1 (1.5625)	0 (0)	0.205	5 (7.8125)	4 (6.25)	2 (3.125)	2 (3.125)	0.454

Syringotropic pattern		1 (1.5625)	1 (1.5625)	0.964	1 (1.5625)	1 (1.5625)	0 (0)	0 (0)	0 (0)	0.767	0 (0)	0 (0)	1 (1.5625)	1 (1.5625)	0.556

Folliculotropic and syrigotropic pattern		2 (3.125)	1 (1.5625)	0.592	0 (0)	1 (1.5625)	2 (3.125)	0 (0)	0 (0)	0.561	1 (1.5625)	1 (1.5625)	0 (0)	1 (1.5625)	0.767

Mucinous change in hair follicle		0 (0)	1 (1.5625)	0.298	1 (1.5625)	0 (0)	0 (0)	0 (0)	0 (0)	0.874	0 (0)	0 (0)	1 (1.5625)	0 (0)	0.422

Granuloma formation		0 (0)	0 (0)	—	0 (0)	0 (0)	0 (0)	0 (0)	0 (0)	—	0 (0)	0 (0)	0 (0)	0 (0)	—

Pigment incontinence		7 (10.9375)	10 (15.625)	0.317	12 (18.75)	2 (3.125)	3 (4.6875)	0 (0)	0 (0)	0.184	4 (6.25)	6 (9.375)	4 (6.25)	3 (4.6875)	0.703

Eosinophil		8 (12.5)	1 (1.5625)	**0.016**	3 (4.6875)	3 (4.6875)	2 (3.125)	1 (1.5625)	0 (0)	0.086	2 (3.125)	3 (4.6875)	1 (1.5625)	3 (4.6875)	0.636

Spongiotic pattern		4 (6.25)	6 (9.375)	0.426	3 (4.6875)	2 (3.125)	5 (7.8125)	0 (0)	0 (0)	0.725	2 (3.125)	2 (3.125)	4 (6.25)	2 (3.125)	0.776

Lichenoid pattern		7 (10.9375)	7 (10.9375)	0.895	8 (12.5)	3 (4.6875)	3 (4.6875)	0 (0)	0 (0)	0.756	4 (6.25)	2 (3.125)	2 (3.125)	6 (9.375)	0.186

Granulomatous pattern		0 (0)	0 (0)	—	0 (0)	0 (0)	0 (0)	0 (0)	0 (0)	—	0 (0)	0 (0)	0 (0)	0 (0)	—

Psoriasiform pattern		18 (28.125)	15 (23.4375)	0.622	15 (23.4375)	8 (12.5)	9 (14.0625)	1 (1.5625)	0 (0)	0.443	9 (14.0625)	7 (10.9375)	12 (18.75)	5 (7.8125)	0.172

Vesiculobullous pattern		0 (0)	0 (0)	—	0 (0)	0 (0)	0 (0)	0 (0)	0 (0)	—	0 (0)	0 (0)	0 (0)	0 (0)	—

Epidermotropism		33 (51.5625)	31 (48.4375)	—	29 (45.3125)	12 (18.75)	21 (32.8125)	1 (1.5625)	1 (1.5625)	—	16 (25)	16 (25)	17 (26.5625)	15 (23.4375)	—

Pautrier's microabscess		13 (20.3125)	17 (26.5625)	0.216	15 (23.4375)	5 (7.8125)	9 (14.0625)	0 (0)	1 (1.5625)	0.635	10 (15.625)	5 (7.8125)	9 (14.0625)	6 (9.375)	0.299

Cytological atypia		20 (31.25)	11 (17.1875)	**0.044**	10 (15.625)	7 (10.9375)	14 (21.875)	0 (0)	0 (0)	0.116	7 (10.9375)	9 (14.0625)	8 (12.5)	7 (10.9375)	0.905

Perivascular superficial infiltrate		13 (20.3125)	10 (15.625)	0.552	9 (14.0625)	7 (10.9375)	7 (10.9375)	0 (0)	0 (0)	0.393	7 (10.9375)	3 (4.6875)	8 (12.5)	5 (7.8125)	0.329

*Note:* The data are presented as the number of cases (%). Chi-square tests were used to calculate the *p* values, which indicate the statistical significance of differences observed between groups for each pattern. Values below 0.05 are considered statistically significant and have been highlighted accordingly.

## Data Availability

The data that support the findings of this study are available from the corresponding author upon reasonable request.
